# Spatial Turing-type Pattern Formation in a Model of Signal Transduction Involving Membrane-based Receptors Coupled by G Proteins

**Published:** 2007-06-06

**Authors:** Chontita Rattanakul, Yongwimon Lenbury, Jonathan Bell, Varanuj Chatsudthipong, Wannapong Triampo, Philip S. Crooke

**Affiliations:** 1 Department of Mathematics, Mahidol University, Bangkok 10400, Thailand; 2 Department of Mathematics and Statistics, UMBC, Baltimore, MD, U.S.A; 3 Department of Physiology, Mahidol University, Bangkok 10400, Thailand; 4 Department of Physics, Mahidol University, Bangkok 10400, Thailand; 5 Department of Mathematics, Vanderbilt University, Tennessee, 37240,U.S.A

**Keywords:** signal transduction, Turing pattern, weakly nonlinear stability, G proteins, membrane based receptors

## Abstract

In this paper, a model of signaling pathways involving G proteins is investigated. The model incorporates reaction-diffusion mechanisms in which various reactants participate inside and on the extra-cellular surface membrane. The messenger molecules may diffuse over the surface of the cell membrane and signal transduction across the cell membrane is mediated by membrane receptor bound proteins which connect the genetically controlled biochemical intra-cellular reactions to the production of the second messenger, leading to desired functional responses. Dynamic and steady-state properties of the model are then investigated through weakly nonlinear stability analysis. Turing-type patterns are shown to form robustly under different delineating conditions on the system parameters. The theoretical predictions are then discussed in the context of some recently reported experimental evidence.

## Introduction

The ability to respond appropriately to signals in the environment is essential for the survival of any organism. A very sophisticated mechanism has therefore evolved for detecting external signals, transducing them into internal signals and eliciting cellular responses. As evidenced by several earlier investigations (Levchenko and Iglesias, 2002; Rapple et al. 2002; Iglesias, 2003; Krishnan and Iglesias, 2003), the cells can sense spatial gradients as temporal changes in receptor occupancy and change certain behavioral patterns in response. Several biological systems have such ability to sense the direction of external chemical sources and respond by transient activation of specific intracellular signaling pathways. This ability to adapt to different levels of external stimuli, so that it is the gradient of signaling molecule rather than the average signal value that determines the response, is a common feature of most chemotactic signaling systems (Levchenko and Iglesias, 2002).

The amoeboid organism *Dictyostelium discoideum* has been widely recognized as a useful model system for the study of chemotaxis (Rapple et al. 2002; Iglesias, 2003). Several recent research reports have proposed and studied mathematical models of signal transduction applied to *Dictyostelium discoideum* and other eukaryotic cells (Levchenko and Iglesias, 2002; Rapple et al. 2002; Krishnan and Iglesias, 2003). In his study of feedback control in intracellular signaling pathways in *Dictyostelium discoideum*, Iglesias (Iglesias, 2003) stated that, for cells to sense and respond to change in their environment, they must first have external sensors on receptors for each of the different stimuli to which it needs to respond. These external messages are then relayed to a series of internal reactants, which in turn trigger key cellular functions. A healthy functioning cell signaling mechanism, termed signal transduction, is essential for the well-being of the life form. Abnormalities of signal transduction pathways have been linked to the development of many serious disorders, such as cancer for example.

Mostly, a cancer cell is a cell that has escaped the controls that maintain its normal differentiated function within the regulatory mechanism of the body (Norman and Litwack, 1997). This therefore underlines the role of signal transduction in the loss of organismic control. In other words, cancer may be viewed as an aberration of the signal transduction process since it derives from a cell that has lost the ability to respond normally to controls from outside, or inside, the cell (Norman and Litwack, 1997). It is thus not surprising that hormones and their receptors are intimately related to carcinogenesis. Many tumors produce ectopical amounts of biologically active hormones that create dysfunctions of the signal transduction process leading to abnormal effects. Various tumors have been shown to secrete ACTH (Adrenocorticotrophic hormone) and to cause hypercortisolism, even when the tumor is undetectably small for many years (Norman and Litwack, 1997).

Additionally, hormones and antihormones are used to treat certain types of cancer. Many cancers are related to the status of hormones in the body. Hormone dependencies of a cell usually are a feature of the presence of the cognate receptor in the cell, while hormone independence becomes a feature of tumor cells that no longer express the appropriate receptor. An avenue for cancer treatment is to remove the grand responsible for the offensive secretion of a hormone. Another avenue would be to utilize appropriate hormones as chemotherapeutic agents. For an example, tamoxifen can interfere with offensive effects of estrogen and result in the inhibition of cellular growth of the tumor. For another example, Vasopressin has been proposed for its potential effect of slowing down the flow of blood that tumors depend on for growth.

Since hormones and their receptors are so closely related to carcinogenesis, better understanding of signal transduction mechanisms have been subject of recent intense research (Norman and Litwack, 1997; Iglesias, 2003). However, signaling pathways are highly nonlinear processes, involving feedbacks and cross-talk among interconnected components, subject to control by many independent events, making clear-cut description highly difficult. New techniques and approaches are needed to understand these complicated networks. According to J. Michael Bishop, “In order to fully understand these pathways, we need a convenient and powerful model to compliment the experimental research” (Iglesias, 2003).

Our goal is thus to extend current understanding of the signal transduction processes by creating a single model explaining adaptation and gradient sensing. Not unlike chemotaxis, subcellular response calls for the cells to detect often exceedingly shallow and changing gradients of extracellular substances and regulating a complex response in accordance with the direction and the value of these gradients. Such highly complex and integrated response needs an explanation and modeling in order to translate biochemical observations and clinical evidence into a set of predictions of dynamical and steady-state properties of the system.

Based on observation and principles of the signal transduction pathways proposed in earlier investigations (Norman and Litwack, 1997; Spiegel, 2000; Levchenko and Iglesias, 2002; Rapple et al. 2002; Iglesias, 2003), we may arrive at a model which describes gradient sensing and adaptation. The resulting model incorporates reaction-diffusion mechanisms in which various reactants participate inside and on the surface of the cell membrane. The messenger molecules diffuse over the extra-cellular membrane surface in two dimensions, while some transport of molecules across cell membrane may take place to a certain extent. Signal transduction across the cell membrane is mediated by membrane receptor bound proteins which connect the genetically controlled biochemical reactions in the cytosol to the production of the second messenger, eliciting desired intracellular responses. Dynamic and steady-state properties of the model are then investigated through the application of weakly nonlinear stability analysis. We show that Turing-type patterns will be formed robustly under different physiological conditions reflected by the system parameters.

The Turing mechanism has often been put forward as a model for certain aspects of morphogenesis (Glansdorff and Prigogine, 1971; Meinhardt, 1982; Koch, 1994; Kondo and Asai, 1995; Sawai et al. 2000; Raunch and Millonas, 2004; Pansuwan et al. 2005) such as pre-patterning in the embryo (Raunch and Millonas, 2004). It has also been a basis for models of self-organization in several other physical systems (Glansdorff and Prigogine, 1971), supported by verifiable observations in a real chemical system (Castets et al. 1990). The investigations involved the possibility of an instability occurring in purely dissipative systems of chemical reactions far from equilibrium and the transport process of diffusion but no hydrodynamic motion. If the system involves two chemical species, commonly termed an activator and an inhibitor, the existence of diffusive instabilities of this sort requires an autocatalytic reaction for the activator and a diffusive advantage for the inhibitor (Turing, 1952). That is, it is necessary that the activator species diffuses significantly more slowly than the inhibitor.

A more recent work by Rauch and Millonas, (2004) showed the formation of Turing-type patterns in trans-membrane signal transduction, by numerical simulations of the models based on their spectrum analysis of the linear stability theory. However, their work involves signalling in the context of a population of cells which are considered as point sources rather than at the subcellular level. Also, a great deal of useful information may be lost through the assumptions of linear stability analysis. In this paper, we are able to predict rhombic and hexagonal planforms in messenger hormone patterns on the cell membrane through nonlinear stability analysis. The theoretical results are then discussed in the context of experimental observations reported in recent literatures.

## G Protein-Coupled Signal Transduction

Since G proteins couple receptors for many hormones to effectors that regulate second messenger metabolism, we shall use heterotrimeric G protein pathways as a model of signal transduction mechanisms. G proteins couple hundreds of receptors for hormones, growth factors, neurotransmitters, odorants and other extracellular ‘first messengers’ to effectors such as adenylyl cyclase (AC), phospholipase *Cβ*, and various ion channels (Spiegel, 2000). In recent years, an increasing number of human disorders, particularly endocrine diseases, have been shown to be caused by mutations in either G protein or G protein-coupled receptors (GPCRs).

G protein-coupled signal transduction involves the following components: GPCRs, the G proteins, G protein-regulated effectors such as AC, and a family of proteins that activates G protein guanosine triphosphatase (GTPase) activity (Spiegel, 2000).

**Figure 1.**
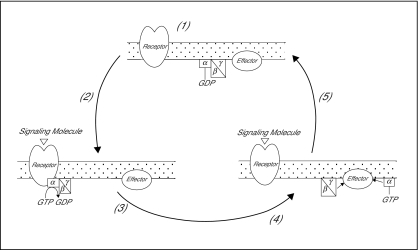
The G protein GTPase cycle (Figure is based on a scheme in the report by A.M. Spiegel (2000)). (1) Synthesis and targeting of components, (2) Receptor activation by signaling molecule, (3) Receptor activation of G protein, (4) G protein subunits-effector interaction and (5) GTPase activity terminates the interaction and returns *α* subunit to inactivated state.

G proteins are heterotrimers composed of three subunits, *α*,*β* and *γ*. The latter two subunits form a tightly, but noncovalently associated functional unit, the *β*/*γ* dimer. In the resting state, the *α*-subunit tightly binds guanosine diphosphate (GDP) and is associated with the *β*/*γ* dimer. When a hormone or other first messenger binds to a receptor, the receptor causes the G protein to exchange GDP for the nucleotide guanosine triphosphate (GTP) which activates the G protein. The GTP-bound *α*-subunit dissociates from the *β*/*γ* dimer. In Figure 1, a schematic description of the pathways, based on a scheme shown in a report by A.M. Spiegel, (2000), is shown, where the arrows between the GTP-bound *α*-subunit and effector and between the *β*/*γ* dimer and effector indicate the interaction between the effector(AC) and the respective subunits. Thus, G proteins are the true signal transducers since they respond to the occupancy of the receptor and modulate the activity of the catalytic subunit of the membrane-bound adenylate cyclase(AC) enzyme (Norman and Litwack, 1997). Under physiologic conditions, effector (AC) activation by G protein subunits is transient and is terminated by the GTPase activity of the *α*-subunit. The latter converts bound GTP to GDP, thus returning the *α*-subunit to its inactivated state with high affinity for the *β*/*γ* dimer which reassociates to again form the heterotrimer (Spiegel, 2000).

The physical interaction of the G protein subunits, with the membrane-bound adenylate cyclase (AC) activates the catalytic activity of AC so that the substrate ATP is converted to produce cyclic adenosine mono-phosphate (cAMP) which functions as a second messenger. Intracellular responses are subsequently elicited. It activates protein kinase A, freeing its active C subunits. These liberated C subunits in turn function as an active kinase to phosphorylate proteins, which thereby amplifies the initial hormonal signal. This amplification effect is an important factor in the signaling pathways and will be incorporated into our model construction later (Equation (2.13)).

To arrive at a model for the signal transduction process, we are guided initially the work of Iglesias, (2003) and let *R* stand for the concentration of regulators in their inactive forms. We may think of these as the inactivated units of adenylate cyclase. The number of activated regulators is then represented by R^*^(*t*). We assume that the activation and inactivation are modulated by a pair of agents *A*, and *I*, respectively. In our particular pathway of interest, the G proteins are the true signal transducers that switch effectors on and off. According to Norman and Litwack, (1997), the *α*-subunits of the G protein take on two functions: *G_αs_* of the amount *A*, which activates the AC, and the active *G_αi_* of the amount *I*, which inhibits the activity of AC. Both *G_αs_* and *G_αi_* are activated by guanosine triphosphate (GTP) and both also functions as a GTPase. The GTPase activity thus endows the G protein with a turn on-off mechanism (Norman and Litwack, 1997). Therefore, *R* transforms to R^*^ according to the following reaction equation.

(2.1) $$\text{R}\overset{\text{A}}{\underset{I}{⇄}}{\text{R}}^{*}$$

Using mass action dynamics, an equation for the above reaction is

(2.2) $$\frac{dR^{*}}{dt}=-k_{-r}\;IR^{*}+k_{r}\;A\;(t)\;R\;(t)$$

Assuming that the total number *R_T_* = *R* + R* of the regulators remains constant, (2.2) becomes

(2.3) $$\frac{dR^{*}}{dt}=-\left[k_{-r}\;I+k_{r}\;A\;(t)\right]R^{*}(t)+k_{r}\;A\;(t)\;R_{T}$$

On the other hand, the activating agent *A* and inhibiting agent *I* are regulated by the external signal which is proportional to the membrane surface concentration *S* of the signaling hormone, such as the external cAMP, giving rise to the following equations:

(2.4) $$\frac{dA}{dt}=-k_{-a}\;A+k_{a}\;S$$

(2.5) $$\frac{dI}{dt\;}=-k_{-i}\;I+k_{i}\;S$$

where the first terms in (2.4) and (2.5) are the rate of removal by natural means and the last terms are rates of their synthesis.

It is commonly assumed (Iglesias, 2003) that in such a process, the activated regulator R^*^ and *A* equilibrate relatively quickly while *I* has the slower dynamics. Using equation (2.3), one finds that R^*^ will equilibrate to the value

(2.6) $$R^{*}=\frac{R_{T}\;A}{A+K_{R}\;I}$$

where

(2.7) $$K_{R}=\frac{k_{-r}}{k_{r}}$$

From setting *Å* = 0 in (2.4), one finds

(2.8) $$A=\frac{k_{a}}{k_{-a}}S$$

which transforms (2.6) into

(2.9) $$R^{*}=\frac{\tilde{k}}{{\tilde{b}}_{1}S+I}S$$

where $\tilde{k}=\frac{R_{T}k_{a}}{K_{R}k{-}_{a}}$ and ${\tilde{b}}_{1}=\frac{k_{a}}{k_{-a}K_{R}}$.

Now, as the subunits of G protein interact with AC aggregates, they activate AC to synthesize intra-cellular cAMP, the level of which is denoted by *C*(*t*), described by the following equation:

(2.10) $$\frac{dC}{dt}=-k_{-c}C+k_{c}R^{*}R^{*}+{k^{\prime }}_{c}$$

in which the first term corresponds to the removal rate. The last two terms correspond to its synthesis, *k'_C_* being the apparent zero order synthesis rate. The second term is the synthesis rate arising from the interaction between the activating subunits *G_αs_* of the G protein of the amount *A* given by (2.8) among the activated catalytic subunits of AC whose amount is R^*^ as given in (2.9).

This enzyme is also found to equilibrate quickly to

(2.11) $$C=\frac{k_{C}}{k_{-C}}R^{*}R^{*}+\frac{{k^{\prime }}_{C}}{k_{-C}}$$

Using (2.8) in (2.11), one obtains

(2.12) $$C=\frac{{\tilde{b}}_{2}S^{2}}{{\left({\tilde{b}}_{1}S+I\right)}^{2}}+K_{C}$$

at equilibrium, where ${\tilde{b}}_{2}=\frac{k_{C}k_{a}\tilde{k}}{k_{-C}k_{-a}}$, and $K_{C}=\frac{k^{\prime }C}{k-C}$.

This cAMP (*C*) in turn acts as a second messenger and amplifies the initial hormone signal *S*(*t,x,y*). Thus, the rate equation for the signaling hormone level at the point (*x,y*) on the extra-cellular membrane-surfaces at time *t* should read as follows.

(2.13) $$\frac{\partial S}{\partial t}=-{\tilde{a}}_{3}S-\frac{{\tilde{b}}_{3}S}{{\tilde{b}}_{4}+S}+k_{S}C(t)+\tilde{\mu }\nabla^{2}S$$

where the first term is the rate of removal by natural means, while the second accounts of its transport through the cell membrane causing its disappearance from the extra-cellular membrane surface. Since it is reasonable to expect this to saturate as the level of hormone increases, the absorption term assumes the form of a Hill-type saturation function, *b*_3_ being the maximum absorption rate and *b*_4_ the half-saturation constant. The third term accounts for the signal amplification arising from the synthesis of cAMP mentioned earlier. We have incorporated the diffusion effect of *S* over the 2 – D cell-membrane surface by the last term in (2.13), where $\nabla^{2}=\frac{\partial^{2}}{\partial x^{2}}+\frac{\partial^{2}}{\partial y^{2}}$, the rate of disappearance through the cell membrane having been accounted for by the second term.

Substituting (2.12) into (2.13), and introducing dimensionless variables $\hat{I}=\frac{I}{{\left[G_{i}\right]}_{T}}$, $\hat{S}=\frac{S}{{\left[G_{s}\right]}_{T}}$, where [*G_i_*]*_T_* and [*G_s_*]*_T_* are the total concentrations per cell of *G_αi_* and *G_αs_*, respectively, in *μmol m*^−2^, we arrive at our model consisting of the following 2 equations

(2.14) $$\frac{\partial \hat{I}}{\partial \tau }=-a_{1}\hat{I}+a_{2}\hat{S}$$

(2.15) $$\begin{array}{l} \frac{\partial \hat{S}}{\partial \tau }=-a_{3}\hat{S}-\frac{b_{1}\hat{S}}{b_{2}+\hat{S}}+\frac{a_{4}{\hat{S}}^{2}}{{\left(a_{5}\hat{S}+\hat{I}\right)}^{2}}\\ \;\;+a_{6}+\mu \nabla^{2}\hat{S} \end{array}$$

where $\tau =\frac{t}{t_{b}}$, *a*_1_ = *t_b_* [*G_i_*]*_T_* *k_−i_*, *a*_2_ = *t_b_* [*G_s_*]*_T_* *k_i_*, *a*_3_ = *t_b_* [*G_s_*]*_T_* *ã*_3_, $a_{4}=\frac{t_{b}{\left[G_{s}\right]}_{T}^{2}}{{\left[G_{i}\right]}_{T}^{2}}$ *b̃_3_*, $a_{5}=\frac{{\tilde{b}}_{1}{\left[G_{s}\right]}_{T}}{{\left[G_{i}\right]}_{T}}$, *a*_6_ = *t_h_k_s_K_c_*, *b*_1_ = *t_b_b̃_3_*, $b_{2}=\frac{{\tilde{b}}_{4}}{{\left[G_{s}\right]}_{T}}$, $\mu =\frac{t_{b}\tilde{\mu }{\left[G_{s}\right]}_{T}}{d^{2}}$, with *t_b_* being the characteristic ligand binding time, and *d* the inner plus outer membrane thickness. Typical values of *t_b_* and *d* are 0.01 s (Erban and Othmer, 2005) and 20 nm (Institute for Biomolecular Design, http://redpoll.pharmacy.ualberta.ca).

In the next section, we shall carry out a weakly non-linear stability analysis on (2.14)–(2.15) in order to show the existence of rhombic and hexagonal planform solutions to our model following the technique discussed by Wollkind et al.(1994) and reviewed by Stephenson and Wollkind (1995).

## Nonlinear Stability Analysis

In order to apply the technique of weakly nonlinear stability theory, we let

(3.1) $$F(\hat{I},\hat{S})=-a_{1}\hat{I}+a_{2}\hat{S}$$

(3.2) $$\begin{array}{l} G(\hat{I},\hat{S})=-a_{3}\hat{S}-\frac{b_{1}\hat{S}}{b_{2}+\hat{S}}\\ \;\;\;\;+\frac{a_{4}{\hat{S}}^{2}}{{\left(a_{5}\hat{S}+\hat{I}\right)}^{2}}+a_{6} \end{array}$$

which transforms (2.14)–(2.15) into

(3.3) $${\hat{I}}_{t}=F(\hat{I},\hat{S})$$

(3.4) $${\hat{S}}_{t}=G(\hat{I},\hat{S})+\mu \nabla^{2}\hat{S}$$

By expanding *F (Î Ŝ)* and *G (Î Ŝ)* into Taylor’s series about the steady state (*I*_0_*,S*_0_) of (3.3) and (3.4) and letting the perturbations *i* ≡ *Î* – *I*_0_, and *s* ≡ *Ŝ* – *S*_0_, we obtain the following system:

(3.5) $$\begin{array}{l} {\left(\begin{array}{c} i\\ S \end{array}\right)}_{t}=\left(\begin{array}{cc} f_{1} & f_{2}\\ g_{1} & g_{2} \end{array}\right)\left(\begin{array}{c} i\\ S \end{array}\right)+\left(\begin{array}{cc} o & o\\ g_{3} & g_{4} \end{array}\right)\left(\begin{array}{c} i^{2}\\ S^{2} \end{array}\right)\\ \;\;\;+\left(\begin{array}{c} o\\ g_{5} \end{array}\right)(iS)+\left(\begin{array}{c} o\\ \mu \nabla^{2}\hat{S} \end{array}\right) \end{array}$$

where

(3.6) $$\begin{array}{l} f_{1}\equiv -a_{1},\;f_{2}\equiv a_{2},g_{1}\equiv -\frac{2a_{4}S_{0}^{2}}{{\left(a_{5}S_{0}+I_{0}\right)}^{3}},\\ g_{2}\equiv -a_{3}-\frac{b_{1}b_{2}}{{\left(b_{2}+S_{0}\right)}^{2}}+\frac{2a_{4}I_{0}S_{0}}{{\left(a_{5}S_{0}+I_{0}\right)}^{3}},\\ g_{3}\equiv \frac{3a_{4}S_{0}^{2}}{{\left(a_{5}S_{0}+I_{0}\right)}^{4}},\\ g_{4}\equiv \frac{b_{1}b_{2}}{{\left(b_{2}+S_{0}\right)}^{3}}+\frac{a_{4}I_{0}(-2a_{5}S_{0}+I_{0})}{{\left(a_{5}S_{0}+I_{0}\right)}^{4}}\text{ and}\\ {\text{g}}_{5}\equiv -\frac{2a_{4}(-a_{5}S_{0}^{2}+2S_{0}I_{0})}{{\left(a_{5}S_{0}+I_{0}\right)}^{4}} \end{array}$$

## A Rhombic Planform Analysis

In order to investigate the possibility of occurrence in our model of rhombic-type patterns, we shall consider a rhombic planform solution of (3.5) of the form (referring the readers to Stephenson and Wollkind, (1995) for the motivation for the choices of functions therein)

(3.7) v˜(x,y,t)∼A1(t)cos(qcx)(11)    +B1(t)cos(qcz)(11)   +A12(t)[v˜2000+v˜2020cos(2qcx)]   +A1(t)B1(t)[v˜1111cos(qc(x+z))    +v˜111(−1)cos(qc(x−z))]   +B12(t)[v˜0200+v˜0202cos(2qcz)]   +A13(t)[v˜3010cos(qcx)    +v˜3030cos(3qcx)]   +A12(t)B(t)[v˜2101cos(qcz)    +v˜2121cos(qc(2x + z))    +v˜212(−1)cos(qc(2x−z))]   +A1(t)B2(t)[v˜1210cos(qcx)    +v˜1212cos(qc(x+2z))    +v˜121(−2)cos(qc(x−2z))]    +B13(t)[v˜0301cos(qcz)    +v˜0303cos(3qcz)]

where $\tilde{v}(x,\;y,\;t)=\left(\begin{array}{c} i(x,y,t)\\ s(x,y,t) \end{array}\right)$, ${\tilde{v}}_{jlmn}=\left(\begin{array}{c} i_{jlmn}\\ s_{jlmn} \end{array}\right)$ and *z* = *x* cos(*φ*) *+ y* sin(*φ*) with the amplitude equations:

(3.8) $$\begin{array}{l} \frac{dA_{1}(t)}{dt}∼\sigma A_{1}(t)-A_{1}(t)[\alpha_{1}A_{1}^{2}(t)\\ \;\;\;\;\;\;+\beta_{1}B_{1}^{2}(t)] \end{array}$$

(3.9) $$\begin{array}{l} \frac{dB_{1}(t)}{dt}∼\sigma B_{1}(t)-B_{1}(t)[\beta_{1}A_{1}^{2}(t)\\ \;\;\;\;\;\;+\alpha_{1}B_{1}^{2}(t)] \end{array}$$

as the zero*^th^* order system of the most dangerous mode (Stephenson and Wollkind, 1995), *α*_1_ and *β*_1_ being the Landau constants to be determined later, as well as *σ.*

In (3.7), we are using the notation *ṽ_jlmn_* for the coefficient of each term in (3.7) of the form $A_{1}^{j}$ (*t*) $B_{1}^{l}$ (*t*)cos (*q_c_* (*mx + nz*)). Please see the work of Stephenson and Wollkind, (1995) for more detail of the technique. On substituting this solution (3.7) into (3.5), we obtain a sequence of vector systems, each of which corresponds to one of these terms. We now catalogue the solutions for the first-order system. In particular, the first order system which corresponds t*o j = m* = 1*, l = n* = 0 is

(3.10) $$\left(\begin{array}{c} \sigma \\ \sigma \end{array}\right)=\left(\begin{array}{cc} f_{1} & f_{2}\\ g_{1} & g_{2} \end{array}\right)\left(\begin{array}{c} 1\\ 1 \end{array}\right)+\left(\begin{array}{c} 0\\ -\mu q_{c}^{2} \end{array}\right)$$

Hence, *σ* = *g*_1_ + *g*_2_ – $\mu q_{c}^{2}$ ≡ *σ*_0_, and *μ* = $\frac{1}{q_{c}^{2}}$(*g*_1_+ *g*_2_− *f*_1_−*f*_2_).

The solutions for the four second-order systems, which can be solved in a straight forward manner, are as follows:

i2000=g3+g42(−g1+g2f1f2),  S2000=−f1f2i2000,i2020=g3+g42(−g1−f1f2(−g2+4μ)),  S2020=(2σ−f1)f2i2020,i1111=(g3+g4)−g1−f1f2[−g2+μ(1+2cos(φ))],  S1111=−f1f2i1111,i111(−1)=(g3+g4)−g1−f1f2[−g2+μ(1−2cos(φ))], and  S111(−1)=−f1f2i111(−1)

From the third-order systems, we can find the Landau constants *α*_1_ and *β*_1_ as

(3.11) $$\begin{array}{l} \alpha_{1}=-\frac{f_{2}}{g_{1}+f_{2}}[(2g_{3}+g_{5})i_{2000}\\ \;\;+(2g_{4}+g_{5})S_{2000}+g_{3}i_{2020}] \end{array}$$

and

(3.12) $$\begin{array}{l} \beta_{1}=-\frac{f_{2}}{g_{1}+f_{2}}[(2g_{3}+g_{5})i_{2000}\\ \;\;+(2g_{4}+g_{5})S_{2000}\\ \;\;+g_{3}(i_{1111}+i_{111(-1)})\\ \;\;+g_{4}(S_{1111}+S_{111(-1)})] \end{array}$$

leaving the derivation to the Appendix.

Having developed these formulae for Landau constants, we now turn our attention to the rhombic planform amplitude equations (3.8)–(3.9) which possess the following equivalence classes of critical points ( $A_{1}^{0}$, $B_{1}^{0}$) when $q_{c}^{2}$ = 1:

(3.13) $$\text{I}:A_{1}^{0}=B_{1}^{0}=0$$

(3.14) $$\text{II}:{\left(A_{1}^{0}\right)}^{2}=\frac{\sigma }{\alpha_{1}},B_{1}^{0}=0$$

(3.15) $$\text{III}:A_{1}^{0}=B_{1}^{0}\text{ with}\;{\left(A_{1}^{0}\right)}^{2}=\frac{\sigma }{\alpha_{1}+\beta_{1}}$$

By assuming that *α*_1_ > 0 and *α*_1_ + *β*_1_ > 0, we investigate the stability of the critical points in (3.13)–(3.15) by seeking a solution of our amplitude equations (3.8)–(3.9) of the form

(3.16) $$A_{1}(t)=A_{1}^{0}+\varepsilon c_{0}\text{exp}(\text{pt})\;+\;O(\varepsilon^{2})$$

(3.17) $$B_{1}(t)=B_{1}^{0}+\varepsilon c_{0}\text{exp}(\text{pt})\;+\;O(\varepsilon^{2})$$

with |***ɛ***| ≪ 1. One finds the following associated roots for *p*.

(3.18) $$\text{I}:p_{1,2}=\sigma ,$$

(3.19) $$\text{II}:\;p_{1}=-2\sigma ,p_{2}=\left(1-\frac{\beta_{1}}{\alpha_{1}}\right)\sigma ,$$

(3.20) $$\text{III}:\;p_{1}=-2\sigma ,p_{2}=\frac{2(\beta_{1}-\alpha_{1})\sigma }{\alpha_{1}+\beta_{1}},$$

which yield the stability criteria for each critical point ( $A_{1}^{0}$, $B_{1}^{0}$) that I is stable for *σ* < 0, II is stable for *σ* > 0, *α*_1_ > 0 and ( $1-\frac{\beta_{1}}{\alpha_{1}}$) < 0, and III is stable for *σ* > 0, *α*_1_ + *β*_1_ > 0 and ( $\frac{\beta_{1}-\alpha_{1}}{\alpha_{1}+\beta_{1}}$) < 0. According to Stephenson and Wollkind (1995), I and II represent the homogeneous and striped states, respectively, while III can be identified with a rhombic pattern possessing characteristic angle *φ*

**Figure 2.**
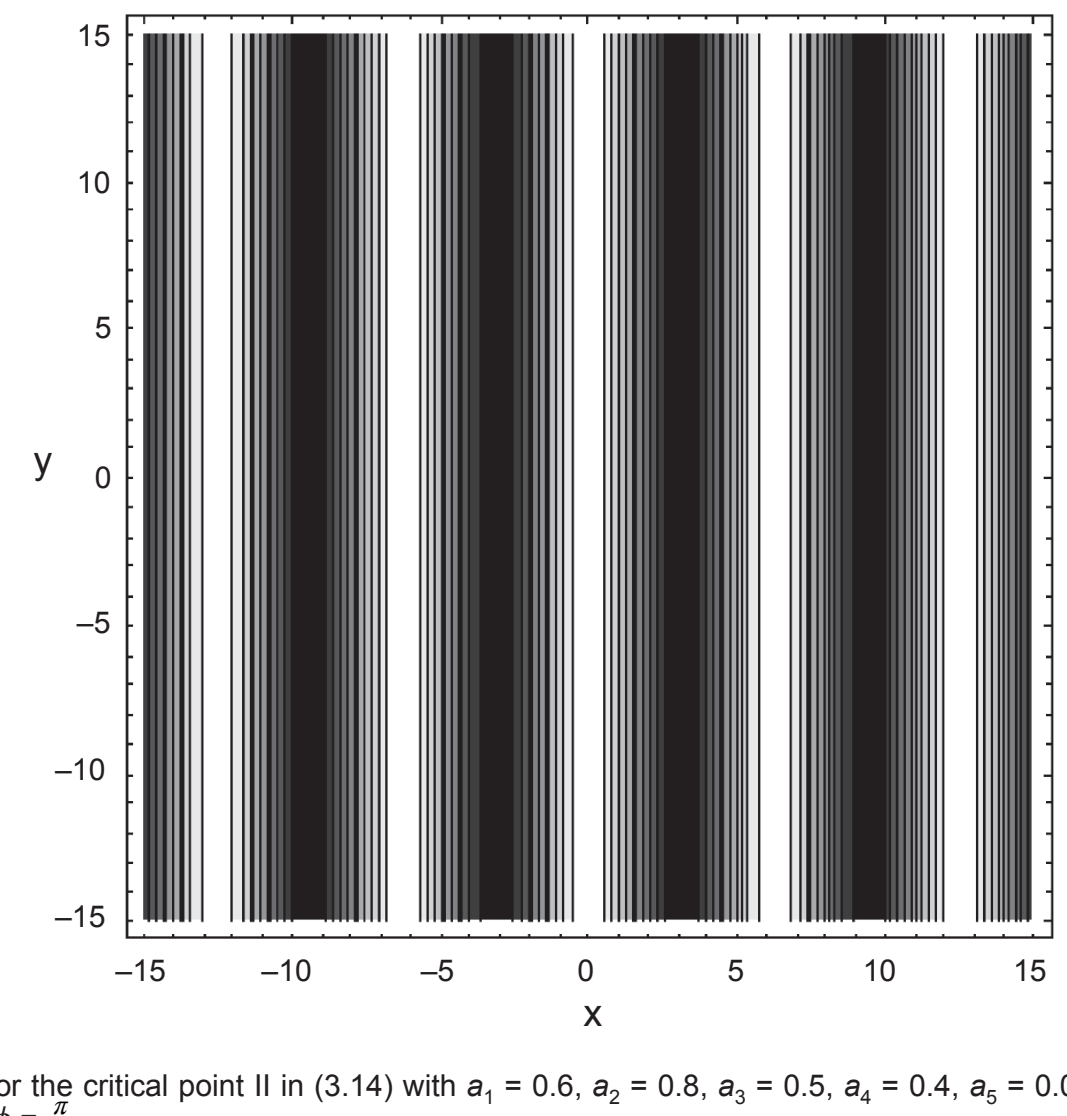
Contour plot of *s* for the critical point II in (3.14) with *a*_1_ = 0.6, *a*_2_ = 0.8, *a*_3_ = 0.5, *a*_4_ = 0.4, *a*_5_ = 0.005, *a*_6_ = 0.001, *b*_1_ = 0.0002, *b*_2_ = 0.55, *μ* = 0.00262, and $\varphi =\frac{\pi }{2}$.

**Figure 3.**
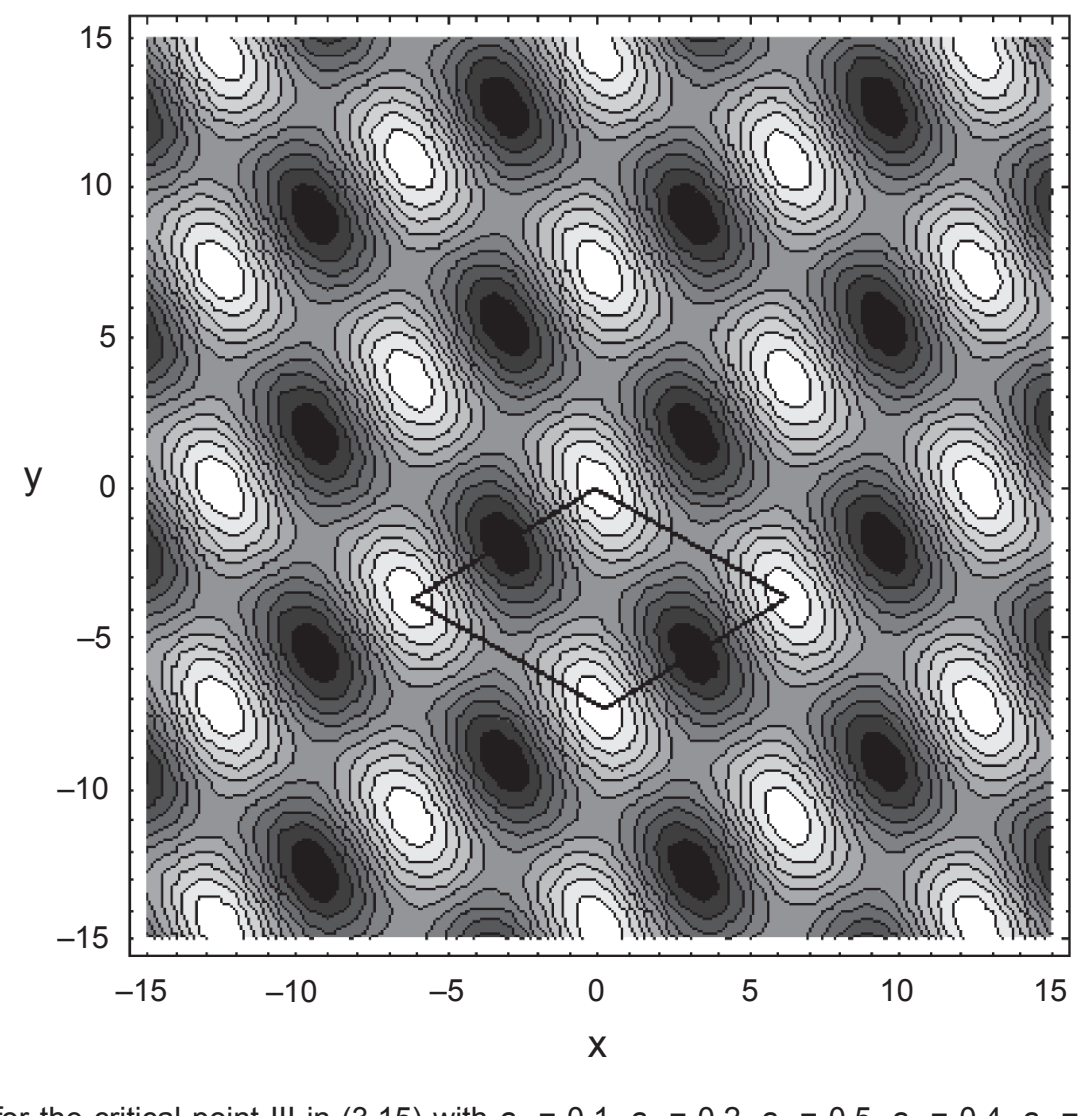
Contour plot of *s* for the critical point III in (3.15) with *a*_1_ = 0.1, *a*_2_ = 0.2, *a*_3_ = 0.5, *a*_4_ = 0.4, *a*_5_ = 0.05, *a*_6_ = 0.001, *b*_1_ = 0.001, *b*_2_ = 1.5, *μ* = 0.0959972, and $\varphi =\frac{\pi }{3}$.

We now investigate the critical points II and III, when stable, of our amplitude equations in relation to Turing patterns of interest. To the lowest order, the solution of the model associated with these critical points is given by the deviation

(3.21) $$S∼A_{1}^{0}\text{cos}\left(\frac{2\pi x}{\lambda_{c}}\right)+B_{1}^{0}\text{cos}\left(\frac{2\pi z}{\lambda_{c}}\right)$$

where *z* = *x* cos(*φ*) *+ y* sin(*φ*) and $\lambda_{c}=\frac{2\pi }{qc}$. The contour plot of this deviation function with $A_{1}^{0}$ > 0 and $B_{1}^{0}$ = 0 relevant to critical point II is shown in the *(x, y*) plane in Figure 2. Clearly, such alternating light and dark parallel bands produced by the critical point II should be identified with a striped Turing pattern as anticipated above. In order to make an analogous interpretation of critical point III, we consider the deviation function (3.21) with $A_{1}^{0}$ = $B_{1}^{0}$ > 0. We generate the contour plot of *s* in Figure 3, which exhibits the rhombic pattern.

## A Hexagonal Planform Analysis

In order to investigate the possibility of occurrence in our model of hexagonal-type patterns, we shall consider a hexagonal planform solution of (3.5) of the form (Stephenson and Wollkind, 1995)

(3.22) $$\begin{array}{l} \tilde{v}(x,y,t)∼A_{2}(t)\text{cos}(q_{c}x)\left(\begin{array}{c} 1\\ 1 \end{array}\right)\\ \;\;\;\;+B_{2}(t)\text{cos}\left(\frac{1}{2}q_{c}x\right)\text{cos}\left(\frac{\sqrt{3}}{2}q_{c}y\right)\left(\begin{array}{c} 1\\ 1 \end{array}\right)\\ \;\;\;+A_{2}^{2}(t)[{\tilde{v}}_{2000}+{\tilde{v}}_{2040}\text{cos}(2q_{c}x)]\\ \;\;\;+A_{2}(t)B_{2}(t)\\ \;\;\;\;[{\tilde{v}}_{1111}\text{cos}\left(\frac{1}{2}q_{c}x\right)\text{cos}\left(\frac{\sqrt{3}}{2}q_{c}y\right)\\ \;\;\;\;+{\tilde{v}}_{1131}\text{cos}\left(\frac{3}{2}q_{c}x\right)\text{cos}\left(\frac{\sqrt{3}}{2}q_{c}y\right)]\\ \;\;\;+B_{2}^{2}(t)[{\tilde{v}}_{0200}+{\tilde{v}}_{0220}\text{cos}(q_{c}x)\\ \;\;\;\;+{\tilde{v}}_{0202}\text{cos}(\sqrt{3}\;q_{c}y)\\ \;\;\;\;+{\tilde{v}}_{0222}\text{cos}(q_{c}x)\text{cos}(\sqrt{3}\;q_{c}y)]\\ \;\;\;+A_{2}^{3}(t)[{\tilde{v}}_{3020}\text{cos}(q_{c}x)+{\tilde{v}}_{3060}\text{cos}(3q_{c}x)]\\ \;\;\;+A_{2}^{2}(t)B_{2}(t)\\ \;\;\;\;[{\tilde{v}}_{2111}\text{cos}\left(\frac{1}{2}q_{c}x\right)\text{cos}\left(\frac{\sqrt{3}}{2}q_{c}y\right)\\ \;\;\;\;+{\tilde{v}}_{2131}\text{cos}\left(\frac{3}{2}q_{c}x\right)\text{cos}\left(\frac{\sqrt{3}}{2}q_{c}y\right)\\ \;\;\;\;+{\tilde{v}}_{2151}\text{cos}\left(\frac{5}{2}q_{c}x\right)\text{cos}\left(\frac{\sqrt{3}}{2}q_{c}y\right)]\\ \;\;\;+A_{2}(t)B_{2}^{2}(t)[{\tilde{v}}_{1200}+{\tilde{v}}_{1220}\text{cos}(q_{c}x)\\ \;\;\;\;+{\tilde{v}}_{1240}\text{cos}({2q}_{c}x)+{\tilde{v}}_{1202}\text{cos}(\sqrt{3}q_{c}y)\\ \;\;\;\;+{\tilde{v}}_{1222}\text{cos}(q_{c}x)\text{cos}(\sqrt{3}\;q_{c}y)\\ \;\;\;\;+{\tilde{v}}_{1242}\text{cos}(2q_{c}x)\text{cos}(\sqrt{3}\;q_{c}y)]\\ \;\;\;+B_{2}^{3}(t)[{\tilde{v}}_{0311}\text{cos}\left(\frac{1}{2}q_{c}x\right)\text{cos}\left(\frac{\sqrt{3}}{2}\;q_{c}y\right)\\ \;\;\;\;+{\tilde{v}}_{0331}\text{cos}\left(\frac{3}{2}q_{c}x\right)\text{cos}\left(\frac{\sqrt{3}}{2}q_{c}y\right)\\ \;\;\;\;+{\tilde{v}}_{0313}\text{cos}\left(\frac{1}{2}q_{c}x\right)\text{cos}\left(\frac{3\sqrt{3}}{2}q_{c}y\right)\\ \;\;\;\;+{\tilde{v}}_{0333}\text{cos}\left(\frac{3}{2}q_{c}x\right)\text{cos}\left(\frac{3\sqrt{3}}{2}q_{c}y\right)] \end{array}$$

with the amplitude equations

(3.23) $$\begin{array}{l} \frac{dA_{2}(t)}{dt}∼\sigma A_{2}(t)-\alpha_{0}B_{2}^{2}(t)\\ \;\;\;\;-A_{2}(t)[\alpha_{1}A_{2}^{2}(t)\\ \;\;\;\;+\alpha_{2}B_{2}^{2}(t)] \end{array}$$

(3.24) $$\begin{array}{l} \frac{dB_{2}(t)}{dt}∼\sigma B_{2}(t)-4\alpha_{0}A_{2}(t)B_{2}(t)\\ \;\;\;\;-B_{2}(t)[2\alpha_{2}A_{2}^{2}(t)\\ \;\;\;\;+\frac{1}{4}(\alpha_{1}+2\alpha_{2})B_{2}^{2}(t)] \end{array}$$

as the zero*^th^* order system of the most dangerous mode (Stephenson and Wollkind, 1995).

In (3.22), we are employing the notation *ṽ_jlmn_* for the coefficient of each term in (3.22) of the form $A_{2}^{j}$ (*t)* $B_{2}^{l}$ (*t*) cos ( $\frac{mq_{c}x}{2}$) cos ( $\frac{n\sqrt{3}q_{c}y}{2}$). Substituting this solution into (3.5) and proceeding in exactly the same manner as in the rhombic planform analysis, we again obtain a sequence of vector systems, each of which corresponds to one of these terms. In particular, the first order system which corresponds to *j* = *m* = 1*,l* = *n* = 0 is

(3.25) $$\left(\begin{array}{c} \sigma \\ \sigma \end{array}\right)=\left(\begin{array}{cc} f_{1} & f_{2}\\ g_{1} & g_{2} \end{array}\right)\left(\begin{array}{c} 1\\ 1 \end{array}\right)+\left(\begin{array}{c} 0\\ -\mu q_{c}^{2} \end{array}\right)$$

Hence, *σ* = *g*_1_ + *g*_2_ − $\mu q_{c}^{2}$ = *σ*_0_, and *μ* = $\frac{1}{q_{c}^{2}}$(*g*_1_ *+ g*_2_ – *f*_1_ – *f*_2_).

There are eight second-order systems, two of which contain the Landau constant *α*_0_. We can write one of them as

(3.26) $$M_{1}{\tilde{v}}_{0220}=\alpha_{0}\left(\begin{array}{c} 1\\ 1 \end{array}\right)+\left(\begin{array}{c} 0\\ \frac{1}{4}(g_{3}+g_{4}+g_{5}) \end{array}\right)$$

where

(3.27) $$\begin{array}{l} M_{1}\equiv \left(\begin{array}{cc} 2\sigma_{0}-f_{1} & -f_{2}\\ -g_{1} & 2\sigma_{0}-g_{2}+\mu q_{c}^{2} \end{array}\right)\\ \;\;=\left(\begin{array}{cc} \sigma_{0}+f_{2} & -f_{2}\\ -g_{1} & \sigma_{0}+g_{1} \end{array}\right) \end{array}$$

Considering the adjoint linear eigenvalue problem of (3.27):

(3.28) $$M_{1}^{T}{\tilde{v}}_{1}^{*}=\sigma_{1}^{*}{\tilde{v}}_{1}^{*}$$

where $\sigma_{1}^{*}$ = *σ*_0_ is an eigenvalue of *M*_1_ and $M_{1}^{T}$, we obtain ${\tilde{v}}_{1}^{*}=\left(\begin{array}{c} g_{1}\\ f_{2} \end{array}\right)$. By taking inner products of (3.26) with ${\tilde{v}}_{1}^{*}$, we find, upon making use of the adjoint property, that

(3.29) $$\begin{array}{l} \sigma_{0}{\tilde{v}}_{0220}\cdot {\tilde{v}}_{1}^{*}=\alpha_{0}\left(\begin{array}{c} 1\\ 1 \end{array}\right)\cdot {\tilde{v}}_{1}^{*}\\ \;\;\;\;\;+\left(\begin{array}{c} 0\\ \frac{1}{4}(g_{3}+g_{4}+g_{5}) \end{array}\right)\cdot {\tilde{v}}_{1}^{*} \end{array}$$

Then, taking the limit as *σ*_0_ → 0 we obtain

(3.30) $$\alpha_{0}=-\frac{f_{2}}{4(g_{1}+f_{2})}(g_{3}+g_{4}+g_{5})$$

The other six second-order systems can be solved in a straight forward manner, yielding solutions as follows:

$$\begin{array}{l} i_{2000}=-\frac{\frac{1}{2}g_{3}+\frac{1}{2}g_{4}+g_{5}}{g_{1}-\frac{g_{2}f_{1}}{f_{2}}},\;S_{2000}=-\frac{f_{1}}{f_{2}}i_{2000},\\ i_{2040}=-\frac{g_{5}+\frac{1}{2}g_{3}+\frac{1}{2}g_{4}}{g_{1}-\frac{f_{1}}{f_{2}}(g_{2}-4\mu )},S_{2040}=-\frac{f_{1}}{f_{2}}i_{2040},\\ i_{0200}=-\frac{g_{3}+g_{4}+g_{5}}{4(g_{1}-\frac{g_{2}f_{1}}{f_{2}})},S_{0200}=-\frac{f_{1}}{f_{2}}i_{0200},\\ i_{0220}=\frac{\alpha_{0}(g_{2}-\mu )-f_{2}(\alpha_{0}+\frac{1}{4}g_{3}+\frac{1}{4}g_{4}+\frac{1}{4}g_{5})}{f_{2}g_{1}-f_{1}(g_{2}-\mu )},\\ \;\;S_{0220}=-\frac{f_{1}i_{0220}+\alpha_{0}}{f_{2}},\\ i_{1111}=\frac{4\alpha_{0}(g_{2}-\mu )-f_{2}(4\alpha_{0}+g_{3}+g_{4}+g_{5})}{f_{2}g_{1}-f_{1}(g_{2}-\mu )},\\ \;\;S_{1111}=-\frac{f_{1}i_{1111}+4\alpha_{0}}{f_{2}},\\ i_{1131}=-\frac{f_{2}(g_{3}+g_{4}+g_{5})}{f_{2}g_{1}-f_{1}(g_{2}-3\mu )},S_{1131}=-\frac{f_{1}}{f_{2}}i_{1131} \end{array}$$

Using the third-order systems, we can find the other two Landau constants *α*_1_ and *α*_2_ as

(3.31) $$\begin{array}{l} \alpha_{1}=-\frac{1}{g_{1}+f_{2}}[\left(2f_{2}g_{3}+f_{2}g_{5}\right)i_{2000}\\ \;\;+f_{2}(2g_{4}+g_{5})S_{2000}\\ \;\;+\left(f_{2}g_{3}+\frac{f_{2}g_{5}}{2}\right)i_{2040}]\\ \;\;-\frac{1}{g_{1}+f_{2}}\left(f_{2}g_{4}+\frac{f_{2}g_{5}}{2}\right)S_{2040} \end{array}$$

and

(3.32) $$\begin{array}{l} \alpha_{2}=-\frac{1}{g_{1}+f_{2}}[8\alpha_{0}g_{1}i_{0220}+8\alpha_{0}f_{2}S_{0220}\\ \;\;+(2f_{2}g_{3}+f_{2}g_{5})i_{0200}\\ \;\;+f_{2}(2g_{4}+g_{5})S_{0200}]\\ \;\;-\frac{1}{g_{1}+f_{2}}[\left(\frac{1}{2}f_{2}g_{3}+\frac{1}{4}f_{2}g_{5}\right)i_{1111}\\ \;\;+\left(\frac{1}{2}f_{2}g_{4}+\frac{1}{4}f_{2}g_{5}\right)S_{1111}\\ \;\;+\frac{3}{8}f_{2}g_{3}i_{1131}]\\ \;\;-\frac{1}{g_{1}+f_{2}}\left(\frac{3}{8}f_{2}g_{4}S_{1131}\right) \end{array}$$

also leaving the detail of the derivation to the Appendix.

Having developed these formulae for Landau constants, we now turn our attention to the hexagonal planform amplitude equations (3.23)–(3.24) which possess the following equivalence classes of critical points ( $A_{2}^{0}$, $B_{2}^{0}$) when $q_{c}^{2}$ =1:

(3.33) $$\text{I}:A_{2}^{0}=B_{2}^{0}=0$$

(3.34) $$\text{II}:{\left(A_{2}^{0}\right)}^{2}=\frac{\sigma }{\alpha_{1}},B_{2}^{0}=0$$

(3.35) $$\begin{array}{l} {\text{III}}^{\pm }:A_{2}^{0}=B_{2}^{0}={\left(A_{2}^{0}\right)}^{\pm }\\ \;\;=\frac{-2\alpha_{0}\pm {\left[4\alpha_{0}^{2}+(\alpha_{1}+4\alpha_{2})\sigma \right]}^{\frac{1}{2}}}{\alpha_{1}+4\alpha_{2}} \end{array}$$

(3.36) $$\text{IV}:A_{2}^{0}=-\frac{4\alpha_{0}}{2\alpha_{2}-\alpha_{1}},B_{2}^{0}=\frac{\sigma -\sigma_{1}}{\alpha_{1}+2\alpha_{2}}$$

where we assume that *α*_1_ > 0, *α*_1_ + 4*α*_2_ > 0, *σ*−_1_ ≡ – $\frac{4\alpha_{0}^{2}}{\alpha_{1}+4\alpha_{2}}$, *σ*_1_ ≡ $\frac{16\alpha_{1}\alpha_{0}^{2}}{{\left(2\alpha_{2}-\alpha_{1}\right)}^{2}}$ and *σ*_2_ ≡ $\frac{32(\alpha_{1}+\alpha_{2})\alpha_{0}^{2}}{{\left(2\alpha_{2}-\alpha_{1}\right)}^{2}}$.

The orbital stability conditions for these critical points can be posed in terms of *σ.* This sort of stability of pattern formation is meant in the sense of a family of solutions in the plane which may interchange with each other but do not grow or decay to a solution type from a different family. Such an interpretation depends upon the translational and rotational symmetries inherent to the amplitude-phase equations. This invariance also limits each equivalence class of critical points to a single member that must be explicitly considered. This line of reasoning was used in our cataloguing of the critical point for the rhombic planform analysis in the previous section as well. Thus, critical point I is stable in this sense for *σ* < 0 while the stability behavior of II and III^±^, which depends upon the signs of *α*_0_ and 2*α*_2_ – *α*_1_ as well, has been summarized in Table 1 under the further assumption that *α*_1_ + *α*_2_ > 0.

We next offer a morphological interpretation of the potentially stable critical points described above relative to the Turing patterns under investigation. To the lowest order, the solution of the model associated with these critical points is given by the deviation function

(3.37) $$S∼A_{2}^{0}\text{cos}(x)+2B_{2}^{0}\text{cos}\left(\frac{1}{2}x\right)\text{cos}\left(\frac{\sqrt{3}}{2}y\right)$$

**Figure 4.**
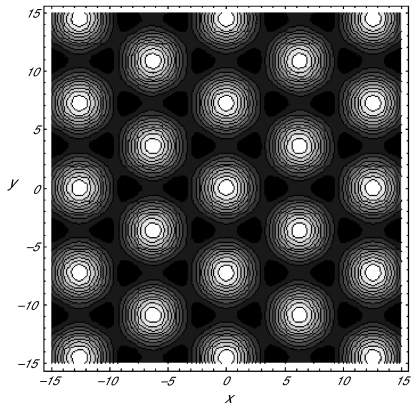
Contour plot of *s* for the critical point III^+^ in (3.35) with *a*_1_ = 0.1, *a*_2_ = 1.5, *a*_3_ = 0.6, *a*_4_ = 0.9, *a*_5_ = 0.001, *a*_6_ = 0.0001, *b*_1_ = 0.5, *b*_2_ =2, *μ* = 0.841495.

**Figure 5.**
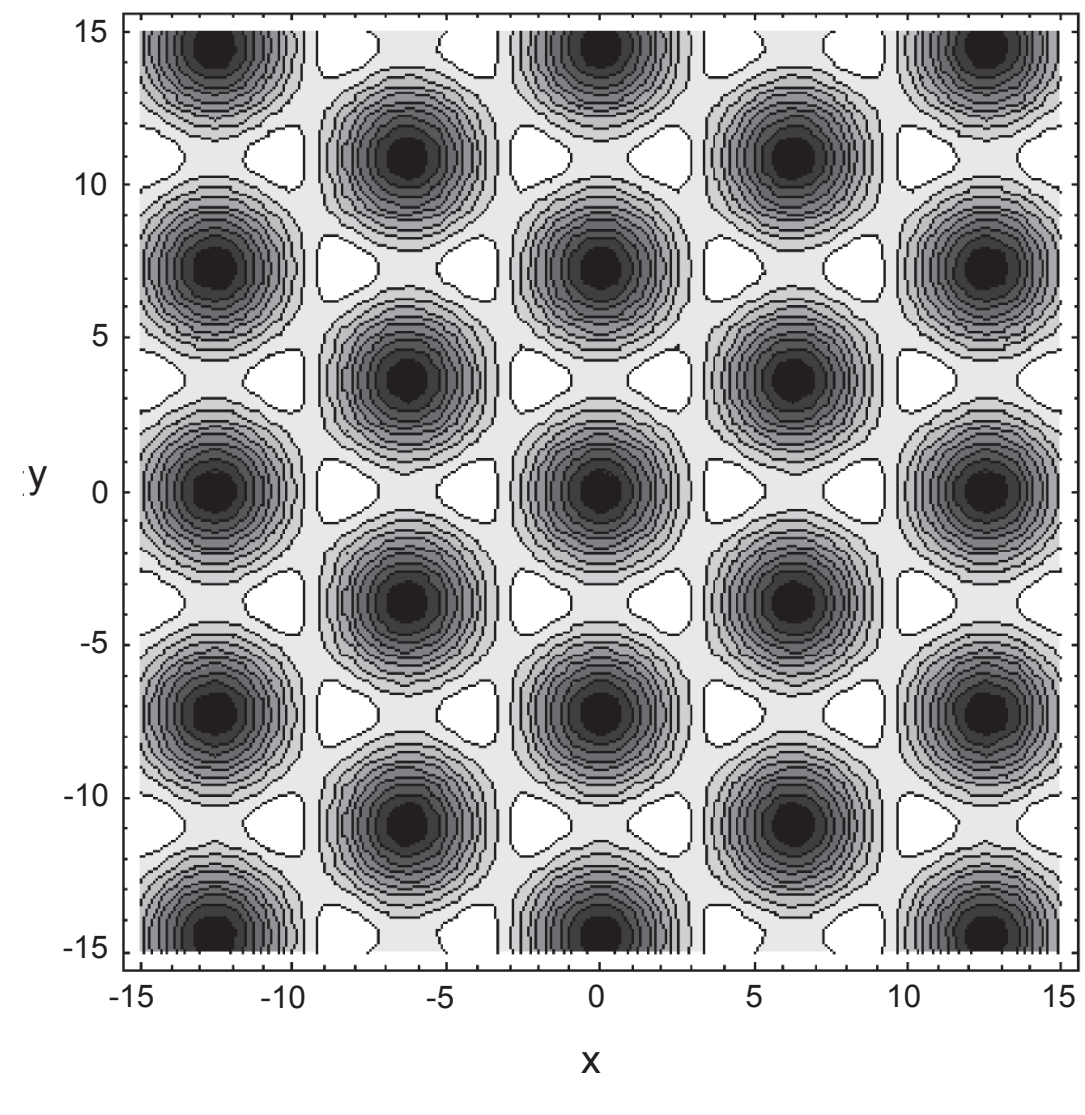
Contour plot of *s* for the critical point III^–^ in (3.35) with *a*_1_ = 0.5, *a*_2_ = 1.5, *a*_3_ = 0.6, *a*_4_ = 0.5, *a*_5_ = 0.00001, *a*_6_ = 0.01, *b*_1_ = 0.95, *b*_2_ = 3, *μ* = 1.13812.

Since ( $A_{2}^{0}$)^+^ > 0 for *α*_0_ ≤ 0 and ( $A_{2}^{0}$)^−^ > 0 for *α*_0_ ≥ 0, we can conclude that the contour plots of (3.37) would have circular elevations at the centers of the hexagonal for critical point III^+^ when stable, and circular depression for III^–^. These contour plots are shown in Figure 4 and Figure 5 for III^+^ and III^–^, respectively. As in analogous plots relevant to our rhombic planform analysis, elevations appear light and depressions dark in these figures. Hence, recalling that the Turing patterns under consideration are classified by their light regions, we identify hexagonal arrays of nets or honeycombs with critical point III^–^ and of spots or dots with critical point III^+^.

## Discussion

Since the values of various kinetic constants are not yet known, we have chosen particular sets of parametric values for illustration purposes, following the example of Krishnan and Iglesias, (2003). For example, to plot the contours in Figure 5, we used the following dimensionless parameters: *a*_1_ = 0.5, *a*_2_ = 1.5, *a*_3_ = 0.6, *a*_4_ = 0.5, *a*_5_ = 0.00001, *a*_6_ = 0.01, *b*_1_ = 0.95, *b*_2_ = 3.0 and *μ* = 1.13812. This dimensionless diffusion coefficient *μ* corresponds to the physical diffusion constant of 2.726 × 10^–10^ *m*^2^*s*^–1^, while the cAMP diffusion constant was estimated to be 2.5 × 10^–10^ *m*^2^*s*^–1^ by Rapple et al.(2002). Our choice of the parametric values took into account several physical features. The activation and deactivation of the inhibitor are more or less of the same order of magnitude. The production rate of cAMP in the absence of the activated regulator in very low so that *a*_6_ ≪ 1. The amplification of the signal by the second messenger is comparable with the removal rate of the signaling hormone from the system.

Comparing the planforms in Figures 4 (quantum dots) and Figure 5 (honeycombs), we observe that quantum dots are expected to occur in the signaling system in which the maximum absorbtion *b*_1_ is not so high. On the other hand, the honeycombs pattern is expected when the maximum rate of disappearance through the membrane of the signaling hormone is relatively high. The values used here are of a comparable order of magnitude as those of the corresponding parameters in the work of Krishnan and Iglesias, (2003). Stripes and rhombic patterns, however result when the rate of diffusion over the membrane surface *μ* and disappearance through the membrane are noticeably lower.

It has only recently been appreciated that G protein coupled receptors (GPCR) and their associated signaling components are not randomly dispersed throughout the plasmalemma. According to Ostrom et al.(2002), expression of *βARs* (*β*-Aderenergic receptors) and AC in several cultured cell models and cardiac muscle cells is enriched in distinct microdomains of the plasma membrane. In these microdomains, one can observe retention of proteins that have particular post-translational modifications (Shaul et al. 1996). Moreover, caveolin in caveolae contains a binding domain that interacts with certain signaling molecules and thereby facilitate localization of signaling molecules in caveolae (Ostrom et al. 2002). Such compartmentation of signaling molecules contradicts the belief that components of GPCR signal transduction are randomly distributed and have extensive mobility in the plasma membrane. On the contrary, these proteins appear to be restricted to plasmalemmal microdomains, probably conductive to rapid and specific signal transduction (Anderson, 1998; Okamoto et al. 1998; Ostrom et al. 2000; Ostrom et al. 2002).

In their recent study, Ostrom et al. (2002) tested the hypothesis that expression and localization of GPCRs and isoforms of AC might be critical determinants of how vascular smooth muscle cells respond to extracellular signals. They observed that localization of AC and the components that influence AC activity might help “tailor” the ability of cells to respond to extracellular and intracellular signals by defining a precise environment in which the second messenger (cAMP) will be generated. In addition, the spatial organization of signaling molecules was found to be a likely important factor in vascular smooth muscle cell regulation, in particular with respect to regulation of cAMP formation which leads to decreased contractile tone, decreased vascular resistance and decreased blood pressure (Ostrom et al. 2002).

Because primary cells in culture can rapidly lose their differentiated phenotype, Ostrom et al.(2002) examined RASMC (rat aortic smooth muscle cells) morphology using transmission electron microscopy. Ultrastructural examination of passage 5 RASMC indicated that these cells possess both morphologic caveolae (light vesicular structures) enriched with *β*-aderenergic receptors, facilitating localization of signaling molecules, which might be identified with stable III^+^ pattern (hexagonal arrays of dots), and morphologic features consistent with contractile phenotype (or filaments) which may facilitate formation of stable pattern of type II (stripes) identified theoretically in the text.

Another recent report by Kriebel et al.(2003) investigated the role of AC localization in regulating the streaming formation in chemotaxis. In their work, the authors presented experimental results concerning the morphological polarization of *Dictyostilium* cells with a cAMP gradient, and showed that local accumulation of AC at the uropod of a cell is required for stream formation. They proposed that the asymmetric distribution of AC provides a compartment from which cAMP may act to amplify the chemical gradient. Thus, this could represent a unique mechanism that cells use to amplify chemotactic responses.

To date, little has been published in terms of pictured spatial distribution of membrane receptors. Using radioactivity-labelled drugs and so-called affinity labels, Peter Gaudeng Waser (1983) has been able to produce binding with high strength or irreversible binding on specific receptors which permitted their isolation and characterization. His electronmicrograph with high resolution shows Acetylcholine receptors in membrane fragments (Waser, 1983) which exhibit hexagonal structure of localization. Other images that show striped or rhombic structures may be found in the work of Unwin et al. (1988) and on http://gingi.uchicago.edu/archr.html. Since signaling molecules are expected to colocalize with their receptors, such pictured receptor complex shown in Waser’s report (1983) provides experimental evidence, to a certain extent, in support of our theoretical predictions that the system of interest may operate, under certain circumstances, in such weakly nonlinear regimes.

## Conclusion

In this paper, we have concentrated on the signal transduction pathways that involve membrane-based receptors which couple to adenylate cyclase. G proteins play a key role in these pathways by activating adenylate cyclase to produce cAMP which functions as a second messenger. Examples of hormones that act via the utilization of a cAMP system are ACTH, GnRH, PTH, and Vasopressin, which are significantly implicated in carcinogenesis.

For some time, various stages of cancers have been classified by means of measurement of receptor levels and their hormone dependence from biopsy specimens. Unfortunately, the indications of measurements of steroid receptor levels are not always absolute and it appears that the ability of receptors to bind steroid does not ensure the complete functioning of the receptors on the cell. Clearly, more research into this area of steroid receptors dysfunction is needed to sharpen prognostic ability. The continued identification of the presence of receptors and their hormone dependence as correlated to their specific functions will greatly enhance prognosis and treatment of such disease.

The result of our mathematical modelling underlines the need for more detailed and precise microscopic study on the spatial distribution of the protein-receptor complexes and the plasmalemma morphology, in the hope of shed more light on the precise manner in which spatial organization of multiple component signal transduction cascades may provide a means to generate signals with high fidelity and efficiency. How the different spatial patterns or gradients described in this paper are related to specific cellular function or dysfunction also remains to be investigated and characterized. More specialized experimental studies are required to provide microscopic data to test hypotheses and establish linkages among the different dynamic activities in the signal transduction cascades. Our model provides a useful context in which to explore the relevance of Turing-type pattern formation to signaling pathways.

## Figures and Tables

**Table 1.** Orbital stability behavior of critical points II and III^±^

*α*_0_	2*α*_2_ – *α*_1_	Stable Structures
+	−, 0	III^−^ for *σ* > *σ*_−1_
+	+	III^−^ for *σ*_−1_ < *σ*< *σ*_2_, II for *σ*> *σ*_1_
0	−	III^±^ for *σ*> 0
0	+	II for *σ*>0
−	+	III^+^ for *σ*_−1_< *σ*< *σ*_2_, II for *σ*> *σ*_1_
−	−, 0	III^+^ for *σ*> *σ*_−1_

## Appendix

### Rhombic Planform

Although there are 10 third-order systems, we need only consider explicitly two specific ones to obtain the Landau constants *α*_1_ and *β*_1_, namely

(7.1) $$\begin{array}{l} M{\tilde{\nu }}_{3010}=\alpha_{1}\left(\begin{array}{c} 1\\ 1 \end{array}\right)\\ +\left(\begin{array}{c} 0\\ (2g_{3}+g_{5})i_{2000}+(2g_{4}+g_{5})S_{2000}+g_{3}i_{2020} \end{array}\right) \end{array}$$

and

(7.2) $$\begin{array}{l} M{\tilde{\nu }}_{2101}=\beta_{1}\left(\begin{array}{c} 1\\ 1 \end{array}\right)+\left(\begin{array}{c} 0\\ (2g_{3}+g_{5})i_{2000}+(2g_{4}+g_{5})S_{2000} \end{array}\right)\\ \;\;\;\;+\left(\begin{array}{c} 0\\ g_{3}(i_{1111}+i_{111(-1)})+g_{4}(S_{1111}+S_{111(-1)}) \end{array}\right)\\ \text{where }M=\left(\begin{array}{cc} 3\sigma_{0}-f_{1} & -f_{2}\\ -g_{1} & 3\sigma_{0}-g_{2}+\mu q_{c}^{2} \end{array}\right)\\ \;\;\;\;=\left(\begin{array}{cc} 2\sigma_{0}+f_{2} & -f_{2}\\ -g_{1} & 2\sigma_{0}+g_{1} \end{array}\right). \end{array}$$

Considering the adjoint linear eigenvalue problem of (7.1)–(7.2) of the form

$$M^{T}{\tilde{v}}^{*}=\sigma^{*}{\tilde{v}}^{*}$$

where *σ*^*^ = 2*σ*_0_ is an eigenvalue of *M* and *M**^T^*, we obtain *ṽ*^*^ = ( $\begin{array}{c} g_{1}\\ f_{1} \end{array}$). By taking inner products of (7.1) and (7.2) with *ṽ*^*^, we find, upon making use of the adjoint property

$$M\tilde{v}\cdot {\tilde{v}}^{*}=\tilde{v}\cdot M^{T}{\tilde{v}}^{*}=\sigma^{*}{\tilde{v}}^{*}\cdot {\tilde{v}}^{*}$$

that

$$\begin{array}{l} 2\sigma_{0}\;{\tilde{v}}_{3010}\cdot {\tilde{v}}^{*}=\alpha_{1}\left(\begin{array}{c} 1\\ 1 \end{array}\right)\cdot {\tilde{v}}^{*}\\ \;+\left(\begin{array}{c} 0\\ (2g_{3}+g_{5})i_{2000}+(2g_{3}+g_{5})S_{2000}+g_{3}i_{2020} \end{array}\right)\cdot {\tilde{\nu }}^{*} \end{array}$$

and

$$\begin{array}{l} 2\sigma_{0}{\tilde{\nu }}_{2101}.{\tilde{\nu }}^{*}=\beta_{1}\left(\begin{array}{c} 1\\ 1 \end{array}\right).{\tilde{\nu }}^{*}\\ \;\;\;\;\;+\left(\begin{array}{c} 0\\ (2g_{3}+g_{5})i_{2000}(2g_{4}+g_{5})S_{2000} \end{array}\right).{\tilde{\nu }}^{*}\\ \;\;\;\;\;+\left(\begin{array}{c} 0\\ g_{3}(i_{1111}+i_{111(-1)})+g_{4}(S_{1111}+S_{111)(-1)}) \end{array}\right) \end{array}$$

Then, taking the limit as *σ*_0_ → 0, we obtain *α*_1_ and *β*_1_ as given in (3.11) and (3.12).

### Hexagonal Planform

Although there are 15 third-order systems, it is necessary for us to consider explicitly only the two specific ones for this planform in order to derive the other two Landau constants *α*_1_, *α*_2_ given below:

(7.3) $$\begin{array}{l} M_{2}{\tilde{v}}_{3020}=\alpha_{1}\left(\begin{array}{c} 1\\ 1 \end{array}\right)\\ +\left(\begin{array}{c} 0\\ (2g_{3}+g_{5})i_{2000}+(2g_{4}+g_{5})S_{2000}+(g_{3}+\frac{1}{2}g_{5})i_{2040} \end{array}\right)\\ \;\;\;\;\;\;\;+\left(\begin{array}{c} 0\\ (g_{4}+\frac{1}{2}g_{5})S_{2040} \end{array}\right) \end{array}$$

and

(7.4) $$\begin{array}{l} M_{2}{\tilde{v}}_{1220}=\alpha_{2}\left(\begin{array}{c} 1\\ 1 \end{array}\right)+8\alpha_{0}{\tilde{v}}_{0220}\\ \;\;\;\;+\left(\begin{array}{c} 0\\ (2g_{3}+g_{5})i_{0200}+(2g_{4}+g_{5})S_{0200} \end{array}\right)\\ \;\;\;\;+(\begin{array}{c} 0\\ \left(\frac{1}{2}g_{3}+\frac{1}{4}g_{4})i_{1111}+(\frac{1}{2}g_{4}+\frac{1}{4}g_{5}\right) \end{array}\\ \;\;\;\;\;S_{1111}+\frac{3}{8}g_{3}i_{1131}+\frac{3}{8}g_{4}S_{1131}) \end{array}$$

where

$$\begin{array}{l} M_{2}\equiv \left(\begin{array}{cc} 3\sigma_{0}-f_{1} & -f_{2}\\ -g_{1} & 3\sigma_{0}-g_{2}+\mu q_{c}^{2} \end{array}\right)\\ \;\;\;=\left(\begin{array}{cc} 2\sigma_{0}+f_{2} & -f_{2}\\ -g_{1} & 2\sigma_{0}+g_{1} \end{array}\right). \end{array}$$

Cosidering the adjoint linear eigenvalue problem of (7.3)–(7.4) of the form

$$M_{2}^{T}{\tilde{\nu }}_{2}^{*}=\sigma_{2}^{*}{\tilde{\nu }}_{2}^{*}$$

where $\sigma_{2}^{*}$ = 2*σ*_0_is an eigenvalue of *M*_2_ and $M_{2}^{T}$, we obtain ${\tilde{v}}_{2}^{*}=(\begin{array}{c} g_{1}\\ f_{2} \end{array})$). By taking inner products of (7.3) and (7.4) with ${\tilde{v}}_{2}^{*}$, we find, upon making use of the adjoint property, that

$$\begin{array}{l} 2\sigma_{0}{\tilde{\nu }}_{3020}\cdot {\tilde{\nu }}_{2}^{*}=\alpha_{1}\left(\begin{array}{c} 1\\ 1 \end{array}\right)\cdot {\tilde{\nu }}_{2}^{*}\\ \;\;\;\;\;+\left(\begin{array}{c} 0\\ (2g_{3}+g_{5})i_{2000}+(2g_{4}+g_{5})S_{2000} \end{array}\right)\cdot {\tilde{v}}_{2}^{*}\\ \;\;\;\;\;+\left(\begin{array}{c} 0\\ (g_{3}+\frac{1}{2}g_{5})i_{2040}+(g_{4}+\frac{1}{2}g_{5})S_{2040} \end{array}\right)\cdot {\tilde{\nu }}_{2}^{*} \end{array}$$

and

$$\begin{array}{l} 2\sigma_{0}{\tilde{\nu }}_{1220}\cdot {\tilde{\nu }}_{2}^{*}=\alpha_{2}\left(\begin{array}{c} 1\\ 1 \end{array}\right)\cdot {\tilde{\nu }}_{2}^{*}+8\alpha_{0}{\tilde{v}}_{0220}\cdot {\tilde{v}}_{2}^{*}\\ \;\;\;\;\;\;+\left(\begin{array}{c} 0\\ (2g_{3}+g_{5})i_{0200}+(2g_{4}+g_{5})S_{0200} \end{array}\right)\cdot {\tilde{v}}_{2}^{*}\\ \;\;\;\;\;\;+(\begin{array}{c} 0\\ \left(\frac{1}{2}g_{3}+\frac{1}{4}g_{4})i_{1111}+(\frac{1}{2}g_{4}+\frac{1}{4}g_{5}\right) \end{array}\\ \;\;\;\;\;\;S_{1111}+\frac{3}{8}g_{3}i_{1131}+\frac{3}{8}g_{4}S_{1131})\cdot {\tilde{v}}_{2}^{*} \end{array}$$

Then, taking the limit as *σ*_0_ → 0 we obtain *α*_1_ and *α*_2_ as given in (3.31) and (3.32).
